# Tumor-targeting *Salmonella typhimurium* A1-R prevents experimental human breast cancer bone metastasis in nude mice

**DOI:** 10.18632/oncotarget.2226

**Published:** 2014-07-16

**Authors:** Shinji Miwa, Shuya Yano, Yong Zhang, Yasunori Matsumoto, Fuminari Uehara, Mako Yamamoto, Yukihiko Hiroshima, Hiroaki Kimura, Katsuhiro Hayashi, Norio Yamamoto, Michael Bouvet, Hiroyuki Tsuchiya, Robert M. Hoffman, Ming Zhao

**Affiliations:** ^1^ AntiCancer, Inc.; San Diego, California USA; ^2^ Department of Surgery; University of California, San Diego; San Diego, California USA; ^3^ Department of Orthopedic Surgery; Kanazawa University Graduate School of Medical Sciences; Kanazawa, Ishikawa, Japan

**Keywords:** breast cancer, bone metastasis, GFP, RFP, bacterial therapy, Salmonella typhimurium A1-R

## Abstract

Bone metastasis is a lethal and morbid late stage of breast cancer that is currently treatment resistant. More effective mouse models and treatment are necessary. High bone-metastatic variants of human breast cancer cells were selected in nude mice by cardiac injection. After cardiac injection of a high bone-metastatic variant of breast cancer, all untreated mice had bone metastases compared to only 20% with parental cells. Treatment with tumor-targeting *Salmonella typhimurium* A1-R completely prevented the appearance of bone metastasis of the high metastatic variant in nude mice (*P* < 0.001). After injection of the highly bone-metastatic breast cancer variant to the tibia of nude mice, *S. typhimurium* A1-R treatment significantly reduced tumor growth in the bone (*P* < 0.001). These data indicated that *S. typhimurium* A1-R is useful to prevent and inhibit breast cancer bone metastasis and should be of future clinical use for breast cancer in the adjuvant setting.

## INTRODUCTION

Bone metastasis is found in more than 80% of patients in advanced stages of breast cancer [1] and is highly treatment resistant and results in extreme pain and high mortality. More effective mouse models and treatment are necessary.

*S. typhimurium*, which is a facultative anaerobe, was previously attenuated with purine and other auxotrophic mutations, for cancer therapy [2]. In a Phase I clinical trial on patients with metastatic melanoma and renal cell carcinoma, the *S. typhimurium* strain tested (VNP20009) was attenuated by lipid A-modified (*msbB*) and purine auxotrophic (*purI*) mutations. VNP20009 was safely administered to patients but colonized the patients’ tumors to a limited extent, perhaps because it was over-attenuated [3].

Another strain of *S. typhimurium*, A1-R, has been developed by our laboratory which has increased antitumor efficacy. *S. typhimurium* A1-R is auxotrophic for Leu-Arg which prevents it from mounting a continuous infection in normal tissues, but does not inhibit tumor targeting and virulence. *S. typhimurium* A1-R has no other attenuating mutations as does VNP20009 and, therefore, may have higher tumor virulence.

The ability to grow in viable tumor tissue may account, in part, for the unique antitumor efficacy of *S. typhimurium* strains [4].

*S. typhimurium* A1-R was able to eradicate primary and metastatic tumors in monotherapy in nude mouse models of prostate, breast, and pancreatic cancer, as well as sarcoma and glioma [4-10]. Tumors with a high degree of vascularity were more sensitive to *S. typhimurium* A1-R, and vascular destruction appears to play a role in *S. typhimurium* A1-R antitumor efficacy [11,12]. Tumor vessel destruction and tumor-growth inhibition was enhanced by primer dosing of *S. typhimurium* A1-R in immunocompetent transgenic mice expressing the nestin-driven green fluorescent protein (ND-GFP), which is selectively expressed in nascent blood vessels [13].

*S. typhimurium* A1-R targeted the Lewis lung carcinoma (LLC) growing subcutaneously in nude mice [14] whereby the bacterially-infected cancer cells greatly expanded and burst and lost viability [15].

We have also shown that *S. typhimurium* A1-R can target chemo-resistant pancreatic cancer stem-like cells [16] and pancreatic cancer patient-derived orthotopic xenograft (PDOX) models [17-19].

In the present study, we demonstrate that *S. typhimurium* A1-R can prevent human breast cancer bone metastasis using a metastatic variant in nude mouse models.

## RESULTS AND DISCUSSION

### Efficacy of *S. typhimurium* A1-R on MDA-MB-435 cells *in vitro*

**Figure 1:**
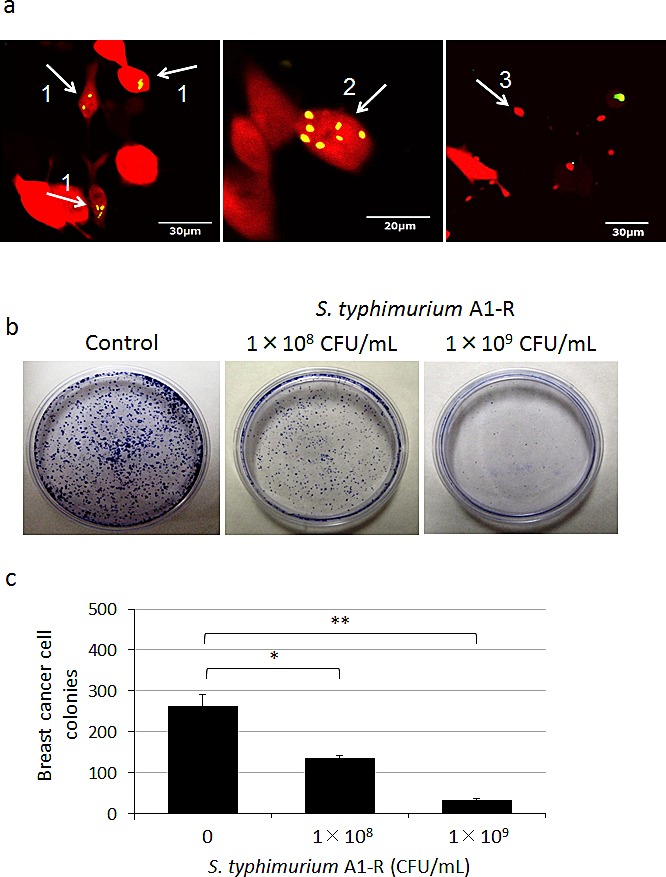
Efficacy of *S. typhimurium* A1-R *in vitro* on breast cancer cells Clonogenic assays were performed as previously described [26]. MDA-MB-435-RFP or MDA-MB-435-GFP cells were planted in 35 mm dishes. MDA-MB-435-RFP cells were observed 24 h after the *S. typhimurium* A1-R treatment with the FV1000 confocal microscope. MDA-MB-435-GFP colonies were fixed with methanol and stained with 1% crystal violet 8 days after *S. typhimurium* A1-R treatment. ImageJ (National Institutes of Health, Bethesda, Maryland, USA) was used to quantify the colonies of the cells. a) *S. typhimurium* A1-R expressing GFP invaded (arrow 1) and replicated intracellularly (arrow 2) in MDA-MB-435-RFP cells. The infected cells fragmented (arrow 3). b) *S. typhimurium* A1-R inhibited proliferation of MDA-MB-435-GFP cells in a dose-dependent manner. c) MDA-MB-435-GFP colony number after A1-R treatment. *p < 0.05, **p < 0.01 compared with the control group.

To determine the efficacy on breast cancer cells, MDA-MB-435 cells, were treated with *S. typhimurium* A1-R for 1 h. Fluorescence imaging demonstrated that *S. typhimurium* A1-R expressing GFP selectively invaded and replicated intracellularly in the MDA-MB-435 cells expressing RFP (Fig. 1a). Clonogenic assays demonstrated that *S. typhimurium* A1-R inhibited proliferation of MDA-MB-435-GFP cells in a dose-dependent manner (Fig. 1).

### *S. typhimurium* A1-R survives in bone marrow

**Figure 2:**
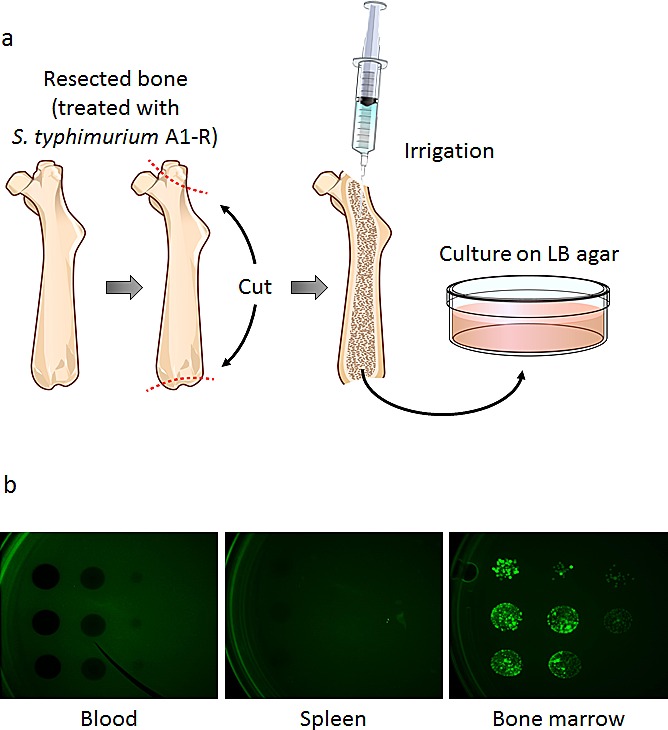
Distribution of *S. typhimurium* A1-R in bone marrow and other organs One, 2, 3, 7, 14, and 28 days after injection of *S. typhimurium* A1-R, the non-tumor-bearing mice were sacrificed and blood, spleen, and bone marrow were harvested and irrigated with PBS. PBS containing *S. typhimurium* A1-R from each tissue was cultured on LB agar. a) Collection of bone marrow. After the resection of the femur, two holes were made in both ends. After irrigation, a portion of the PBS was cultured on LB agar for 24 hours. b) Colony formation of GFP-expressing *S. typhimurium* A1-R obtained from blood, spleen, and bone marrow which were harvested 14 days after *S. typhimurium* A1-R treatment. *S. typhimurium* A1-R fluorescent colonies were imaged with the OV100 Small Animal Imaging System.

After injection of *S. typhimurium* A1-R (5×10^7^ CFU, i.v.) to non-tumor-bearing nude mice, blood, spleen, and bone marrow were cultured on Luria-Bertani (LB) agar (Fig. 2a). The presence of *S. typhimurium* A1-R was confirmed by GFP-expressing colony formation 24 hours after culture (Fig. 2b). These results showed that *S. typhimurium* A1-R survived for 2 weeks in bone marrow, compared to only 3 days in spleen. Blood had no colony formation on LB agar.

### *In vivo* selection of highly metastatic breast cancer cells

**Figure 3:**
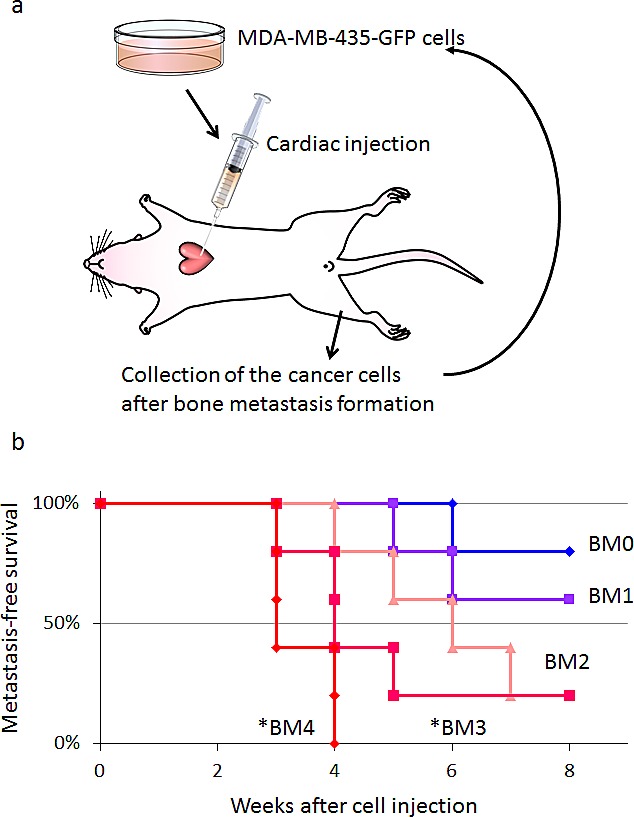

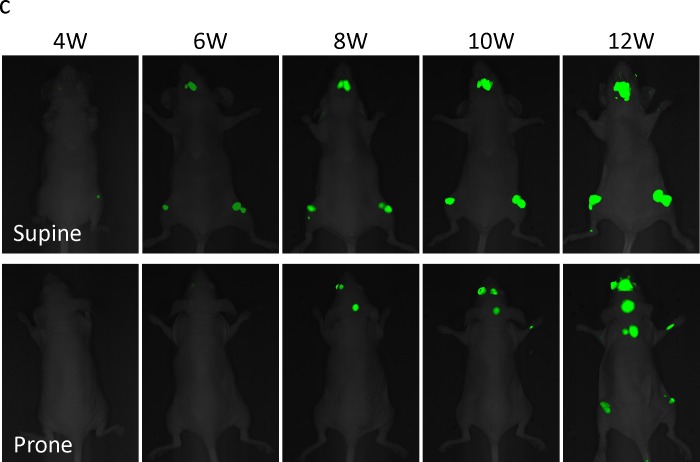
*In vivo* selection of highly brain-metastatic cancer cells a) Schematic representation of the in vivo cyclic selection process for high bone-metastatic variants. MDA-MB-435-GFP cells were inoculated into the left cardiac ventricle of nude mice. Six weeks after injection, experimental bone metastases were detected by fluorescence imaging. Cancer cells were isolated from bone metastases and re-inoculated in the left ventricle after expansion in culture. Cells isolated from the second round of metastases were expanded in culture and re-inoculated in the left ventricle for a total of 4 cycles. b) Kaplan-Meier curves for metastasis-free survival of mice inoculated in the left ventricle with MDA-MB-435-GFP-BM1-4 cells compared with the parental MDA-MB-435-GFP cells (BM0) (*p < 0.05). c) Time-course imaging of breast cancer bone metastasis. MDA-MB-435-GFP-BM4 cells were inoculated into the left cardiac ventricle of nude mice. Fluorescence imaging visualized the progression of metastases in multiple bones including skull, femur, and vertebra.

MDA-MB-435-GFP cells formed experimental bone metastases after injection in the left cardiac ventricle injection of nude mice (Fig. 3a). Bone metastases become detectable by fluorescence six weeks after inoculation of parental MDA-MB-435-GFP cells (2×10^5^), and appeared in 20% of inoculated mice. High metastatic variants obtained after 1−4 cycles of *in vivo* selection were progressively termed MDA-MB-435-GFP-BM1, -BM2, -BM3, and -BM4. After 4 cycles of selection, the highly metastatic cell line MDA-MB-435-GFP-BM4 was obtained (Fig. 3b). MDA-MB-435-GFP-BM4 cells generate detectable bone metastases 3-4 weeks after inoculation, in 100% of the mice inoculated (Fig. 3c).

### Efficacy of *S. typhimurium* A1-R therapy on experimental bone metastasis

**Figure 4:**
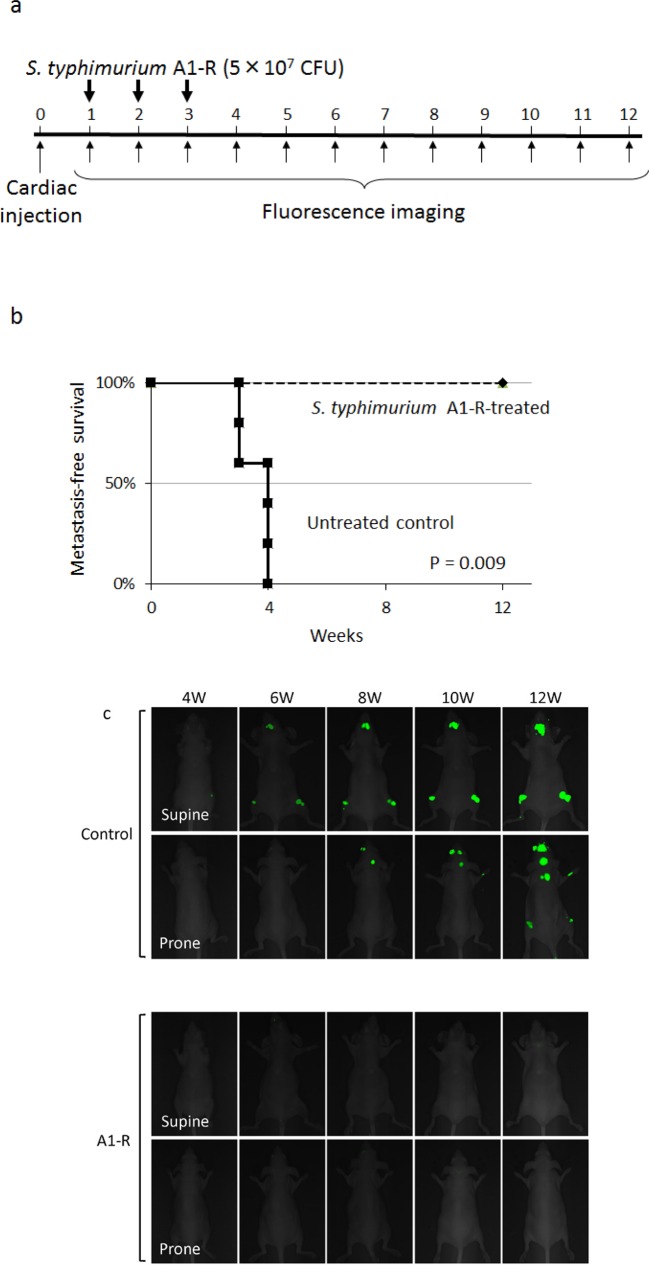
Efficacy of *S. typhimurium* A1-R on experimental bone metastasis after cardiac injection a) Study protocol. MDA-MB-435-GFP-BM4 cells were injected into the left ventricle in nude mice (n=10). On day 7, 14, and 21, *S. typhimurium* A1-R (5×10^7^ CFU/mouse, n) were administered to mice (n=5). Fluorescence imaging was performed every week. b) Metastasis-free survival of mice treated with *S. typhimurium* A1-R or untreated controls was determined using the Kaplan-Meier method with log-rank test. c) Time-course fluorescence imaging of breast cancer bone metastasis with or without treatment of *S. typhimurium* A1-R. MDA-MB-435-GFP-BM4 cells were inoculated in the left cardiac ventricle of nude mice. Fluorescence imaging visualized the progression of metastases in multiple bones including skull, femur, and vertebrae in control mice. In contrast, there was no fluorescence in the mice treated with *S. typhimurium* A1-R.

Nude mice (n=10) were injected in the left ventricle with MDA-MB-435-GFP-BM4 cells (2×10^5^). One week after cardiac injection, mice (n=5) were treated 3 times with weekly i.v. injection of *S. typhimurium* A1-R (Fig. 4a). Four weeks after cardiac injection of MDA-MB-435-GFP-BM4 cells, fluorescence imaging showed experimental metastatic bone metastasis in all of the untreated control mice (Fig. 4b). In contrast, no *S. typhimurium* A1-R-treated mouse had bone metastasis during the 12-week observation period (*P* = 0.009) (Fig. 4c).

### Efficacy of *S. typmimurium* A1-R on breast cancer cell growth in the tibia

**Figure 5:**
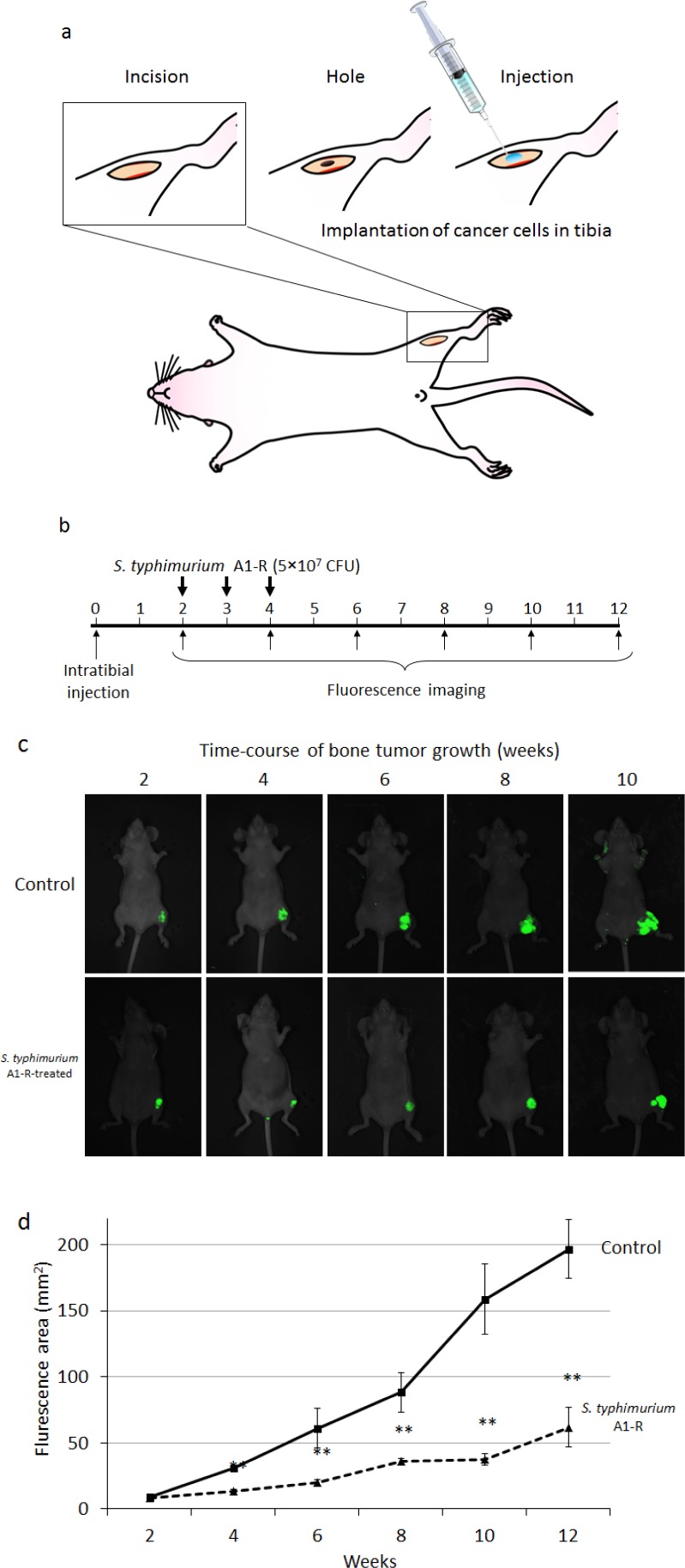
a) Experimental schema of approximately 5 mm midline skin incision was made to expose the tibial tuberosity MDA-MB-435-BM4 cells (2×10^5^) in 5 μl Matrigel (BD Bioscience, San Jose, CA) were injected in the intramedullary cavity of the tibia. b) Study protocol. Two weeks after intratibial injection, fluorescence imaging was performed to confirm the growing GFP-expressing tumor using the iBOX Scientia Small Animal Imaging System (UVP LLC, Upland, CA, USA). Mice (n=5) (treatment group) were administered *S. typhimurium* A1-R (5×10^7^ CFU, i.v.) once a week for 3 weeks. The remaining mice (untreated control group, n=5) were administered the same volume of PBS. Fluorescence imaging was performed on treated and untreated mice. GFP fluorescent areas were recorded every 2 weeks for 12 weeks using the iBOX. c) Time-course imaging of the GFP-expressing bone tumors in *S. typhimurium* A1-R therapy treated mice and untreated mice. d) Fluorescence area of bone tumors in untreated control and *S. typhimurium* treated mice. Data are expressed as the mean ± SE. Differences between groups were analyzed with the Student's *t*-test. **p < 0.01 compared with the untreated control group.

MDA-MB-435-GFP-BM4 cells (5×10^5^) were injected into the intramedullary cavity of the tibia in nude mice (n=12) (Fig. 5a). Two weeks after intratibial injection of cancer cells, six mice were treated three times with weekly i.v. *S. typhimurium* A1-R (Fig. 5b). Fluorescence imaging demonstrated that the control mice had rapid tumor growth of the tibial tumor. In contrast, the *S. typhimurium* A1-R-treated mice had slow growth (Fig. 5c). The fluorescent tumors area of the untreated control mice and *S. typhimurium* A1-R-treated mice 10 weeks after bacterial therapy was 196.6 ± 22.1 mm^2^ and 61.8 ± 15.0 mm^2^, respectively (*P* < 0.001) (Fig. 5d). The bone marrow was observed with an FV1000 confocal microscope to confirm the presence of GFP-expressing cancer cells.

The present study demonstrates that *S. typhimuium* A1-R has and could significantly inhibit or prevent bone metastasis. These results indicate a promising approach to a currently highly treatment-resistant disease.

## MATERIALS AND METHODS

### Cell culture

MDA-MB-435-GFP cells and MDA-MB-435-RFP cells were generated as previously described [20-25]. Cells were maintained in Dulbecco's modified Eagle's medium high glucose supplemented with 10% FBS + 200 μg/mL G418 (Gibco). Cells were subcultured for at least 3 passages before harvesting at their linear growth phase (approximately 70-80% confluent) for cardiac injection of 2×10^5^ cells.

### Preparation of *S. typhimurium* A1-R

GFP-expressing *Salmonella typhimurium* A1-R bacteria (AntiCancer Inc., San Diego, CA, USA) were grown overnight on LB medium (Fisher Sci., Hanover Park, IL, USA) and then diluted 1:10 in LB medium. Bacteria were harvested at late-log phase, washed with PBS, and then diluted in PBS [4, 5].

### *S. typhimurium* A1-R killing of breast cancer cells in vitro

MDA-MB-435-GFP or MDA-MB-435-RFP cells were planted in 35 mm dishes (2×10^3^). *S. typhimurium* A1-R-GFP was grown in LB medium and added to the cancer cells (1×10^8^ or 1×10^9^ CFU/dish). After 1 h incubation at 37°C, the cells were rinsed and cultured in medium containing gentamycin sulfate (100 μg/ml) to kill external but not internal bacteria. Invasion and destruction of MDA-MB-435-RFP cells by *S. typhimurium* A1-R-GFP was visualized with the FV1000 confocal microscope. Eight days after treatment with *S. typhimurium* A1-R, MDA-MB-435-GFP colonies were fixed in methanol and stained with 1% crystal violet as previously described [26]. ImageJ (National Institute of Mental Health, Bethesda, Maryland, USA) was used to quantify the colonies of the cells.

### Distribution of *S. typhimurium* A1-R in blood, spleen, and bone marrow tissue

Tumor-free nude mice were injected with *S. typhimurium* A1-R (5×10^7^ CFU, i.v.). On days 1, 2, 3, 7, 14, and 28 after *S. typhimurium* A1-R GFP injection, femurs were taken from 3 mice and irrigated with PBS. Spleens were minced and mixed with PBS, as was blood. The PBS, including *S. typhimurium* A1-R from each tissue, was plated on LB agar containing 50 μg/mL ampicillin to identify *S. typhimurium* A1-R in each tissue. Fluorescent *S. typhimurium* A1-R colonies were observed with the OV100 Small Animal Imaging System (Olympus Corp., Tokyo, Japan).

### *In vivo* cycle selection of highly metastatic breast cancer cells

MDA-MB-435-GFP cells were harvested from subconfluent cell culture plates, washed with PBS, and resuspended in PBS. Cells (2×10^5^) were injected into the left cardiac ventricle of female nude mice using a 27G needle. Mice were anesthetized with a ketamine mixture (10 μl ketamine, HCL, 7,6 μl xylazine, 2.4 μl acepromazine maleate and 10 μl H_2_O) before injection. A successful injection was characterized by the pumping of arterial blood into the syringe. Development of bone metastases was initially monitored with an Illumatool imaging system (Lightools Research, Encinitas, CA, USA). To isolate cancer cells from the bone metastasis, mice were sacrificed, and the affected bones were excised. Both ends of the bones were cut open. A one ml syringe with a 27G needle was filled with PBS and inserted into one end of the bone. Mouse bone marrow cells as well as cancer cells were forced out from the other end by applying pressure to the syringe. Cells were collected by centrifugation and washed once with PBS before being cultured at 37°C. After two weeks of culture, a pure population of human cancer cells was obtained as confirmed by fluorescence imaging. After 4 cycles of this procedure, we obtained a highly metastatic cells line, termed MDA-MB-435-GFP-BM4. Time-course imaging of the mice after cardiac injection of MDA-MB-435-GFP-BM4 demonstrated progression of multiple metastases to bone including skull, femur, and vertebrae.

### Efficacy of S. typhimurium A1-R therapy on early-stage experimental bone metastasis

MDA-MB-435-GFP-BM4 cells (2×10^5^) were injected intracardially in nude mice. One week after injection, mice (n=5) (treatment group) were administered *S. typhimurium* A1-R (5×10^7^ CFU, i.v.) once a week for 3 weeks. The remaining mice (control group) were administered the same volume of PBS. To evaluate metastasis-free survival, GFP-expressing lesions were initially observed using the Illumatool every week. Metastatic bone lesions were also imaged every two weeks using the iBOX Scientia Imaging System (UVP LLC, Upland, CA, USA). Metastasis-free survival was defined as the time from cardiac injection of cancer cells to the time of detection of bone metastases with the Illumatool. At the end of the follow-up, the metastases were excised and the bone marrow was washed with PBS to confirm the presence of GFP-expressing cancer cells.

### Efficacy of *S. typhimurium* therapy on breast cancer cell growth in the tibia

A midline skin incision (5 mm) was made just below the knee joint to expose the tibial tuberosity (Fig. 6). Matrigel (5 μL) (BD Bioscience, San Jose, CA) and MDA-MB-435-GFP-BM4 cells (5×10^5^) were co-injected into the intramedullary cavity of the tibia with a 0.5 mL 28 G latex-free insulin syringe (0.5 mL 28 G) (TYCO Health Group LP, Mansfield, MA). The skin was closed with a 6-0 suture. Two weeks after injection, fluorescence imaging was performed to confirm the GFP-expressing tumor was growing, using the iBOX. Nude mice (n=5) were administered *S. typhimurium* A1-R (5×10^7^ CFU, i.v.) once a week for 3 weeks. The remaining mice (untreated control group) were administered the same volume of PBS. Fluorescence imaging was performed on treated and untreated mice, and GFP-expressing areas were recorded every 2 weeks for 12 weeks using the iBOX.

### Statistical analysis

Data showing comparisons between two groups were assessed using the Student's *t*-test. Comparisons among more than two groups were assessed using analysis of variance (ANOVA). The Kaplan-Meier method was used for bone metastasis-free survival and log-rank test was used for statistical significance of the difference between the two groups. Differences were considered significant when p < 0.05. Data are expressed as mean ± SEM. Statistical analyses were performed with EZR (Saitama Medical Center, Jichi Medical University).
